# Exploring Short-Term Responses to Changes in the Control Strategy for *Chlamydia trachomatis*

**DOI:** 10.1155/2012/803097

**Published:** 2012-06-03

**Authors:** James Clarke, K. A. Jane White, Katy Turner

**Affiliations:** ^1^Centre for Mathematical Biology, University of Bath, Bath BA2 7AY, UK; ^2^School of Social and Community Medicine, University of Bristol, Canynge Hall, 39 Whatley Road, Bristol BS8 2PS, UK

## Abstract

Chlamydia has a significant impact on public health provision in the developed world. Using pair approximation equations we investigate the efficacy of control programmes for chlamydia on short time scales that are relevant to policy makers. We use output from the model to estimate critical measures, namely, prevalence, incidence, and positivity in those screened and their partners. We combine these measures with a costing tool to estimate the economic impact of different public health strategies. Increasing screening coverage significantly increases the annual programme costs whereas an increase in tracing efficiency initially increases annual costs but over time reduces costs below baseline, with tracing accounting for around 10% of intervention costs. We found that partner positivity is insensitive to changes in prevalence due to screening, remaining at around 33%. Whether increases occur in screening or tracing levels, the cost per treated infection increases from the baseline because of reduced prevalence.

## 1. Introduction

Infection with *Chlamydia trachomatis* is a problem for infected individuals and health services. Chlamydia is the most prevalent sexually transmitted infection (STI) in the UK [1], with a large proportion of cases asymptomatic, and untreated infection can lead to serious complications for men and women. Men can develop epididymitis as a result of infection, while untreated chlamydia in women can cause pelvic inflammatory disease (PID), ectopic pregnancy, and tubal factor infertility [1]. A randomised control trial in the USA found that screening for chlamydia reduced the incidence of PID by 56% [2]. Treatment of chlamydia is straightforward, and front-line therapy is a single dose of azithromycin which is effective at clearing chlamydia and has recently been approved by the Medicines and Healthcare products Regulatory Agency (MHRA) for use without prescription [3]. The problems caused by chlamydia are estimated to cost the National Health Service (NHS) in England over *£*100 m every year [3] and cause morbidity in some individuals.

In England in 2003 the National Chlamydia Screening Programme (NCSP) was set up; central to its mission was reducing the onward transmission of genital chlamydia infection and preventing sequelae through early detection [1]. The NCSP targets young persons under 25 years outside of sexual health clinics, screening individuals opportunistically when they visit other health services [4]. Prevalence of chlamydia is highest among young people, with those under 25 most likely to be infected [5]. Screening an individual is a quick, easy, noninvasive process, and if someone is found to be infected, an effort is made to offer a screen to their previous sexual partners [4]. Thus contact tracing forms an integral part of the NCSP, working alongside opportunistic screening. In 2009 the NCSP tested 16% of young people aged 15–24 years [4] compared to a target of 17% for that period. Longer-term goals are to achieve a screening coverage of 35%–50% [3], although it is uncertain how long it will take to achieve these goals since increasing national screening coverage is complex. However, there are uncertainties about the effects and the cost effectiveness of the programme [6]. The question that we wish to address is the following: how will achieving these targets impact on prevalence, incidence, and positivity within the time scales used to determine policy decisions (taken to be up to five years)?

Different modelling approaches have been used for chlamydia and other similar infections such as gonorrhea. Two aspects stand out as important in STI modelling: heterogeneity in the sexual activity of the population [7] and the strong influence of social structure on epidemic dynamics [8]. Unlike airborne diseases, STIs can only be spread as a result of clearly defined sexual interaction, and hence random mixing assumptions used in more basic models fail to capture an important aspect of STI transmission. Previous work by Anderson and May [9], Hethcote and Yorke [10], and others [11, 12] has used mean-field ordinary differential equations (ODEs) to look at the spread of HIV/AIDS, gonorrhea, and chlamydia. In these models heterogeneity between individuals has been incorporated through the use of a “core” group which is small in size but high in sexual activity, sustaining disease in a population where it would otherwise be eradicated. Although easier to analyse, these models are too simple to model contact tracing effectively.

Studies informing public health policy have often used large individual-based stochastic simulations [7, 13–15]. In these simulations it is possible to track the behaviour of each individual and hence implement control policies exactly as conceived. However, these models also require a large number of parameters to be estimated, and in the case of chlamydia these are not always known accurately. For example, transmission probabilities and natural clearance rates are subject to some uncertainty since they cannot be studied easily. The three models compared by Kretzschmar et al. all assume a transmission probability per sex act, even though previous work [16] has shown that the number of sexual exposures to an infected partner does not correlate with infection status. The relative effectiveness of screening and contact tracing has been examined for a general disease on random networks [17], and it has also been shown that in a general SIS model there is a critical prevalence below which contact tracing becomes cost effective [18].

Pair approximation equations have been successful in bridging the gap between stochastic network simulations and mean-field ODE models [8, 19–22]. They model how the number of pairs of a certain type varies with time, as well as the number in each class of individuals. The equations for the number of pairs involve the number of triples, but instead of forming the triple equations the system is usually closed at the level of pairs using a moment closure approximation [23, 24]. These models aim to capture the influence that network structure has on the spread of disease but without actually modelling specific individuals. Pair models are appropriate here because they have a lot fewer parameters than individual-based models but can still capture the network behaviour necessary for disease transmission and contact tracing.

Of particular relevance, Eames and Keeling [8] and House and Keeling [25] modelled the spread of STIs in a closed population to explore the efficacy of contact tracing. They investigated the relationship between critical tracing efficiency (efficiency needed to prevent invasion of the disease) and the basic reproductive number *R*_0_, finding that *R*_0_ can be used to estimate critical tracing efficiency and that clustering in the network improves the efficacy of contact tracing.

The aim of this study is to use pair approximation equations to capture the essence of network dynamics in a set of ODEs and use these to measure the impact of different control strategies for chlamydia on realistic, short time scales. We investigate outcomes linked to positivity, prevalence, incidence, and cost, showing how control programmes may affect directly observable quantities.

## 2. Methodology

**Figure 1:**
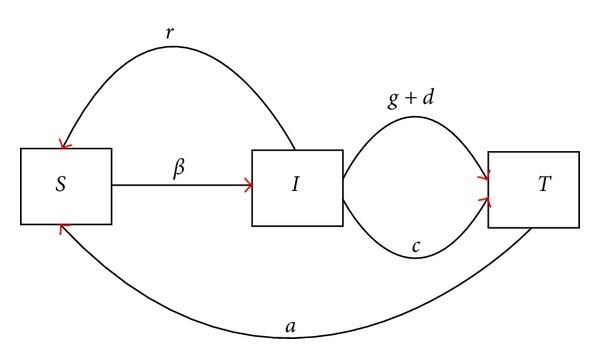
Flowchart of the model system.

Our model is developed from previous work [8, 25] restricted to a target population in the 16–25-year age bracket. The population is assumed to be of constant size *N* following [26]; this is a reasonable assumption since recovered individuals return to the susceptible pool (possibly following a period of immunity) and since we are only concerned with a five-year time frame. Susceptible individuals become infected either if they are in a partnership with an infected individual or if they are in a partnership with a susceptible individual but have contact external to that partnership with an infected individual. The rate at which infection happens is *β*. Once infected, an individual may move into the treatment class either because they develop symptoms naturally at rate *d* or as a result of being screened at rate *g*. Infected individuals may clear infection without treatment and return to the susceptible class which they do at rate *r* < *d*. Partner notification (PN) also means that infected individuals may move into the treated class if they are in a partnership with an individual in the treatment class and they do so at a rate *c*. Assuming that treatment is successful, individuals return to the susceptible class at rate *a*. This gives rise to the model system:

(1) $$\begin{array}{cc} \left[\dot{S}\right] & =-\beta \left[SI\right]+a\left[T\right]+r\left[I\right],\\ \left[\dot{I}\right] & =\beta \left[SI\right]-\left(g+d+r\right)\left[I\right]-c\left[IT\right],\\ \left[\dot{T}\right] & =\left(g+d\right)\left[I\right]+c\left[IT\right]-a\left[T\right], \end{array}$$

where [*S*], [*I*], and [*T*] denote the number of individuals in each of the classes susceptible, infected, and treated, respectively, and where [*SI*] denotes the number of partnerships in which one individual is susceptible and the other infected, and so forth. A flowchart of the system is shown in Figure 1. To fully specify the model, we need to write down the time evolution equations for each pair; these are given in Appendix A where it can be seen that they involve the parameter *k* which measures the average number of partnerships per individual. Baseline parameter estimates are derived from published data and are given in Table 1. The infection rate across an *S*-*I* link (*β*) is estimated by keeping all other parameters constant and then setting *β* to achieve a steady-state prevalence of 8%.

We investigate how changes in the control parameters (*c* and *g*), assumed to be independent of each other, impact on prevalence, incidence, and positivity. Each measure is calculated for the first, third, and fifth year after a step change in the control parameters. In the definitions below *t*_*i*_ is the day at which the *i*th year begins: for example, if a step change in *c* is at *t* = 0, then *t*_3_ = 2∗365 = 730.

**Prevalence**

This is given by the proportion of individuals infected and is calculated using the value at the end of the year in question by

(2) $$\begin{array}{c} \frac{\left[I\right]+\left[T\right]}{N}\left(t_{i}\right). \end{array}$$

**Positivity**

We consider two measures of positivity.

- *Individual positivity*. Here we assume that people undergoing treatment in the *T* class will not be screened, so this is different from population prevalence. Positivity is perhaps the most important quoted statistic for a screening programme such as the NCSP. It is calculated as
  (3) $$\begin{array}{c} \frac{1}{365}\overset{t_{i+1}}{\underset{t_{i}}{\int }}\frac{\left[I\right]}{\left[S\right]+\left[I\right]}dt \end{array}.$$
- *Partner positivity*. The equivalent “positivity” measure for contact tracing is defined as an average over a year (as above) of the proportion of contacts of *T* individuals who are infected, given by
  (4) $$\begin{array}{c} \frac{1}{365}\overset{t_{i+1}}{\underset{t_{i}}{\int }}\frac{\left[IT\right]}{\left[ST\right]+\left[IT\right]}dt. \end{array}$$
  Once again the assumption is that if the partner of a *T* individual is also a *T*, then no effort will be expended in tracing them. This measure is important for analysing how correlated infectious individuals are with other infected people and how much more likely you are to find an infected individual through contact tracing instead of screening.

**Incidence**

This is defined as the number of new cases in a given year and is given as cases per 10000 person-years. It is important to ensure a good match between model and real incidence, as well as estimated prevalence, as incidence gives an indication of disease turnover which will impact on the efficacy of control measures [13]. It is calculated as

(5) $$\begin{array}{c} \overset{t_{i+1}}{\underset{t_{i}}{\int }}\beta \left[SI\right]dt \end{array}.$$

The number of partners per individual, *k*, was set using figures calculated in [27], which is based on estimates from NATSAL 2000. This is the National Survey of Sexual Attitudes and Lifestyles, a survey carried out in Great Britain between 1999 and 2001, which looked at the sexual behaviour of people aged between 16 and 44 [30]. The screening parameter *g* is calculated from the percentage of the population who is screened over a period of one year. This allows the parameter to be compared directly with figures published by the NCSP. If a proportion *s* of the population is screened per year, *g* can be calculated using

(6) $$\begin{array}{cc} s & =1-e^{-365g} \end{array}.$$

Tracing success is measured as *c*/*a*, the proportion of partners traced. 

The rate of naturally developing symptoms (*d*) is given by assuming that some small fractions of those becoming infected go on to be symptomatic (20% in this case) and that if they do, then they do so quickly with an average time spent infected of 30 days [12, 14]. 

We obtain values for the measures listed above from numerical simulations of the model system. We run the system to equilibrium and then make a step change in either *c* or *g* and record the new values for each measure one, three and five years following that change. More realistically there would be a gradual change over a period of time, which means that our results provide an upper bound on the possible impact. 

In addition, we use a published costing tool [31] to explore the economic impact of changing levels of screening or partner notification. Since resources are limited, decisions must be made on how to share these between activities linked to screening individuals and activities focussed on contact tracing. We explore this interdependence by numerically solving the system to find the tracing efficiency that gives the specified target prevalence after 3 years, for different screening coverages. For a specified screening coverage, we calculate the level of tracing needed to reach target prevalence. All numerical results in this work were obtained using MATLAB. 

## 3. Results

In each of the following figures we show the baseline case (where the system is at steady state with annual screening coverage of 16% and tracing efficiency of 30%) along with the results from changing the control parameters. 

**Figure 2:**
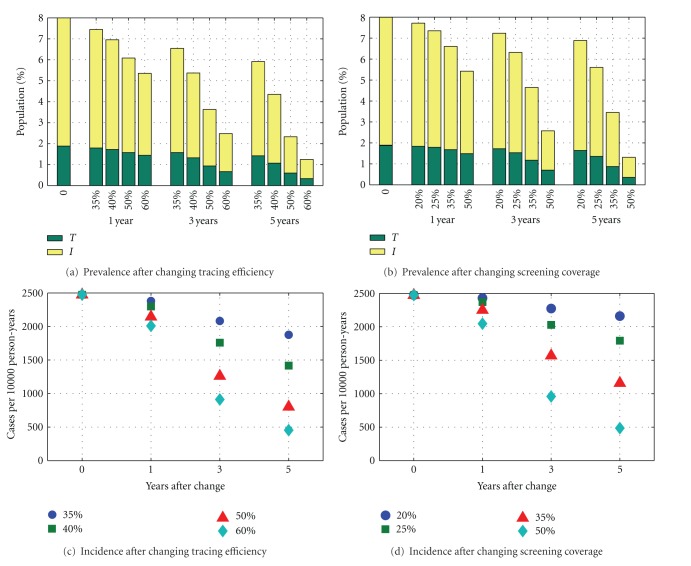
Prevalence (top) and incidence (bottom) after specified changes to tracing efficiency (left) and annual screening coverage (right) in parameters where baseline prevalence is 8%. Prevalence figures are broken down into those in the *I* and *T* classes.

In Figure 2 changes in prevalence (Figures 2(a) and 2(b)) and incidence (Figures 2(c) and 2(d)) are shown, where screening coverage is increased to 20%, 25%, 35%, or 50% and tracing efficiency is increased to 35%, 40%, 50%, or 60%. Assuming that screening and partner notification rates can be varied independently, our results are consistent with previous work, but the novelty here lies in our focus on short-term behaviours. In every situation the size of the treatment class remains essentially unchanged for at least three years, after which it decreases in size. Screening and partner notification both have similar impacts, causing reductions in overall prevalence and incidence.

Baseline incidence levels in Figure 2 compare favourably with those in large individual-based models. Kretzschmar found that incidences in the models being compared were between 500 and 4000 cases per 10000 person-years [13], while Batteiger recently found that incidence in a real world study was around 3400 cases [32]. The figure here of 2500 cases is most similar to that given by the HPA model [13, 14], which has figures between 1000 and 2000 depending on age range. As expected, the incidence follows a similar pattern to that for prevalence, with screening levels at 50% per year or tracing efficiency at 60% able to significantly reduce the number of new cases in the population after five years. An increase in contact tracing has more of an effect over the long term compared to screening, although over a shorter period, the effects are similar (Figure 2). 

**Figure 3:**
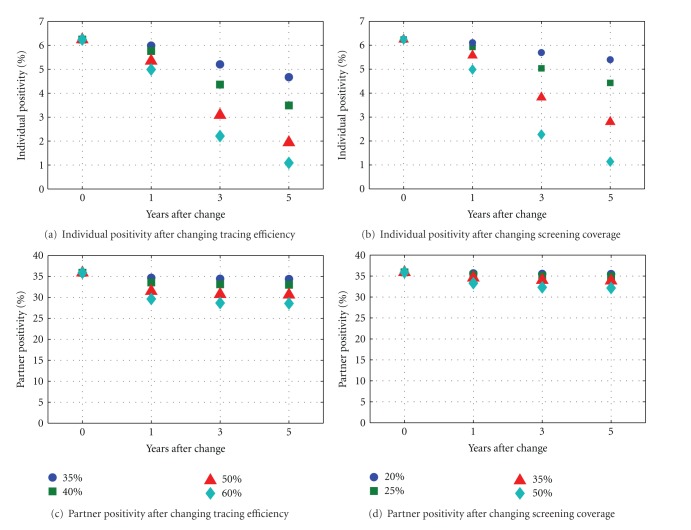
Individual (top) and partner (bottom) positivity after changes to tracing efficiency (left) and annual screening coverage (right) for a baseline prevalence of 8%.

Figure 3 shows the values of individual positivity and partner positivity in a similar way, except that values are shown as points instead of bars instead of bars. Positivity (both individual and partner) provides the clearest indication of where screening and contact tracing will be most effective. Figure 3 shows individual (Figures 3(a) and 3(b)) and partner positivity (Figures 3(c) and 3(d)), and as expected individual positivity is similar to prevalence since only a relatively small proportion of people are in the treatment class at any one time, and it is only these people who will not be available for a random screening programme. Partner positivity is a different story; screening and contact tracing do not massively affect the chance of finding an infected individual in a partnership with somebody receiving treatment (partner positivity), with values starting at 36% and remaining close to 30% even after five years of increased control efforts. This contrasts with individual positivity that starts at a baseline value of just over 6% and then falls away with amounts depending on how much control is being implemented. This is important for comparing the effectiveness of the two measures, since the lower the prevalence, the greater the relative chance of finding another infected individual through contact tracing instead of screening. As prevalence increases, the effectiveness of screening will be increased while that of contact tracing will remain roughly similar. 

### 3.1. Financial Costs

Using the tool developed by Turner et al. [31], preliminary cost analysis is presented in Table 2. This highlights the dependency between screening and partner notification, showing that as prevalence decreases, cost per infection will increase. This would be expected for a situation where there is a large control effort (with large cost) that continues regardless of prevalence. We focus soley on the output generated by the tool in question in order to demonstrate that dynamic models can be combined with existing tools to improve their accuracy. 

Increasing tracing efficiency to 40% incurs a cost increase of around *£*1 m in the first year, with a cost per treated infection of *£*660.92 compared to the baseline case of *£*633.26 per infection. After three years, costs have been reduced to below the baseline value, but now the cost per treated infection is *£*847.77 because prevalence has been reduced from the baseline value of 8% to 5.3%. Increasing screening coverage to 25% represents a large increase in total annual costs of about *£*25 m, with a cost per infection treated in the first year of *£*665.89. This is larger than the cost per infection in the first year when tracing efficiency is increased, and prevalence is higher in the screening case (7.3% compared to 7% for tracing). After three years with increased screening, coverage total costs are still about *£*25 m above the baseline case, with a cost per infection of *£*775.29. This is less than the cost per infection after three years of improved tracing, but prevalence is 6.3% compared to 5.3% with tracing. Overall, the cost effectiveness of increasing contact tracing efficiency compared to screening coverage is clear, with greater reductions in prevalence for less cost. 

### 3.2. Interdependence

**Figure 4:**
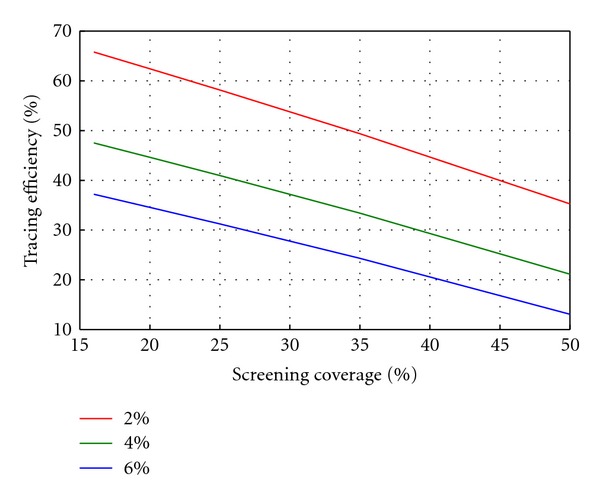
Tracing efficiency required for specified screening coverage in order to achieve target prevalence of 2%, 4%, and 6% after three years.

In reality, the amount of screening and/or partner notification that one is able to carry out will depend on how limited resources are allocated between the two. In Figure 4 we show how different combinations of screening and partner notification will achieve the same target prevalence after three years. Each line shows combinations of tracing efficiency and screening coverage that will achieve a prevalence of 2%, 4%, or 6% after three years, from a baseline of 8% prevalence. 

### 3.3. Robustness of Results

A sensitivity analysis was performed on the model, with outputs given by varying model parameters *a*, *d*, *r*, and *k* by ± 10%. For figures and more details, see Appendix B. Our results prove robust to these changes in parameters, with the most significant effect seen in the impact on contact tracing when *k*, the average number of connections per individual, is varied. This is as one would expect, since tracing is heavily dependent on the network structure, but even so the variation is small and does not affect the conclusions of this work. 

## 4. Discussion

Using a deterministic set of pair approximation equations we have explored the effect of increasing screening or contact tracing levels over time scales relevant to policy makers. This relatively simple model has been applied to chlamydia infection and treatment, with the model parameters largely taken from the literature. Although theoretical work has been done on a similar model [25], we have studied the short-term impact of changes in control strategy and looked at how the system behaves dynamically. Partner positivity has been defined in terms of this model, and we have demonstrated that this measure stays relatively unchanged even when prevalence and individual positivity are decreasing. Partner positivity is insensitive to changes in screening coverage or tracing efficiency, remaining at around 33%. This is important when considering how best to target a control programme, since our results, along with previous work [18], suggest that there is a critical prevalence below which contact tracing is advisable. 

In addition, we have linked our dynamic results with a previously published costing tool which is used by health care providers [31]. This highlights the cost effectiveness of increasing tracing efficiency instead of screening coverage when the underlying model is not static. Combining this analysis with the interdependence results in Figure 4 provides a means for estimating the most cost-effective strategy combination for achieving a target prevalence in a certain time frame. Since the costs of partner notification are an order of magnitude less than screening, and PN has a greater chance of success, it is important that any control programme pursues the partners of any index case. Further work will explicitly model the resource limitations inherent in a public health programme and identify how best to distribute the resources across screening and tracing. 

Efforts have been made to use accurate parameters based on available data, but there is still a great deal of uncertainty around some critical quantities, for example, how fast individuals recover without any treatment or how much immunity someone has after clearing infection naturally. However, our sensitivity analysis has shown that the conclusions remain valid under a wide range of parameter values. There is some scope for extending this approach to a heterogeneous population by having individuals with different numbers of partners, either by having them incorporated into one population and modelling every permutation of different types of connection or by having several different homogeneous populations with links between. 

In the meantime, by using the approach detailed here, comparisons can be made that will, at the very least, provide qualitative and order of magnitude quantitative information about changes in intervention strategies for chlamydia. 

## Figures and Tables

**Figure 5:**
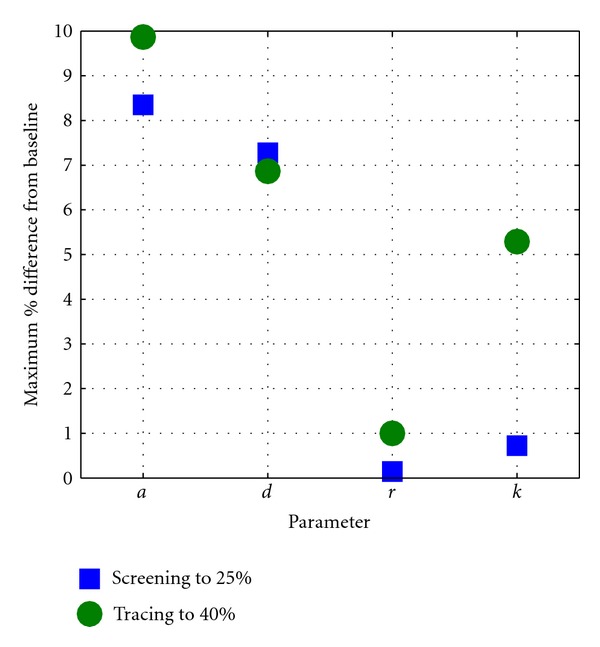
Maximum *relative* % difference from baseline for each parameter, when screening coverage is increased to 25% or tracing efficiency to 40%.

**Table 1:** Baseline parameters with ranges used in sensitivity analysis (where appropriate), descriptions and sources used for estimation.

Parameter	Value	Range	Description	Source
*β*	0.01425 days^−1^	[0.01136, 0.01923]	Infection rate across an *S*-*I* link with 8% baseline prevalence	Set for 8% prevalence
*k*	2.07	[1.863, 2.277]	Number of partners per individual	[13, 27]
*a*	0.02857 days^−1^	[0.0257, 0.0314]	Recovery rate from treatment to susceptible class	[7, 13, 14, 28]
*c*	0.00857 days^−1^	N/A	Rate of contact tracing across *I*-*T* link with 30% tracing efficiency	[4, 29]
*g*	4.78 × 10^−4^ days^−1^	N/A	Screening rate assuming 16% coverage per year	[3, 4]
*d*	0.00667 days^−1^	[0.0060, 0.0073]	Rate of developing symptoms naturally	[12] & See text
*r*	0.00231 days^−1^	[0.0021, 0.0025]	Rate of natural clearance without developing symptoms	[12]
*N*	10000	N/A	Population size	—

**Table 2:** Annual costs of screening and partner notification (PN) when screening coverage is increased to 25% and tracing efficiency to 40%. Costs are shown for one year and three years after the change. The first row shows the case at the 8% baseline steady state.

—	Annual cost of screening (*£*)	Annual cost of PN (*£*)	Total annual cost (*£*)	Cost per infection treated (*£*)	Prevalence (%)
Baseline	42,951,600	4,319,000	47,270,600	633.26	8
Tracing efficiency to 40%—1 year	42,951,600	5,387,140	48,338,740	660.92	7
Tracing efficiency to 40%—3 years	42,951,600	4,086,796	47,038,396	847.77	5.3
Screening coverage to 25%—1 year	67,111,875	6,421,901	73,533,776	665.89	7.3
Screening coverage to 25%—3 years	67,111,875	5,442,289	72,554,164	775.29	6.3

## Appendices

### A. Model Equations

The full system of model equations is

(A.1) $$\begin{array}{cc} \left[\dot{SS}\right] & =2a\left[ST\right]+2r\left[SI\right]-2\beta \left[SSI\right],\\ \left[\dot{SI}\right] & =\beta \left(\left[SSI\right]-\left[ISI\right]-\left[SI\right]\right)-c\left[SIT\right]+a\left[IT\right]\\ & \;-\left(g+d+r\right)\left[SI\right]+r\left[II\right],\\ \left[\dot{ST}\right] & =\left(g+d\right)\left[SI\right]+a\left[TT\right]-\beta \left[TSI\right]+c\left[SIT\right]-a\left[ST\right]\\ & \;+r\left[IT\right],\\ \left[\dot{II}\right] & =2\beta \left(\left[SI\right]+\left[ISI\right]\right)-2c\left[IIT\right]-2\left(g+d+r\right)\left[II\right],\\ \left[\dot{IT}\right] & =c\left(\left[IIT\right]-\left[IT\right]-\left[TIT\right]\right)-\left(g+d+r+a\right)\left[IT\right]\\ & \;+\beta \left[TSI\right]+\left(g+d\right)\left[II\right],\\ \left[\dot{TT}\right] & =2\left(c+g+d\right)\left[IT\right]+2c\left[TIT\right]-2a\left[TT\right]. \end{array}$$

These equations contain terms involving the number of triples of certain types, where [*ABC*] represents a *C* individual connected to an *A*-*B* pair. Equations for pairs involving two individuals of the same type (e.g., [*TT*]) contain factors of two because they represent links in both directions across (*T*-*T*) partnerships, whereas other pair equations (e.g., *S*-*I*) only represent one direction (not  *I*-*S*) but their counterparts are not modelled explicitly because they evolve in exactly the same way. To close the system we must express triples in terms of pairs; to do this we assume that pairs are independently binomially distributed so that

(A.2) $$\left[ABC\right]=\frac{k-1}{k}\frac{\left[AB][BC\right]}{\left[B\right]},$$

where *k* is as described in Table 1. Individual class sizes can be recovered from the pair structure using the following expressions:

(A.3) $$\begin{array}{cc} \left[S\right] & =\frac{1}{k}\left(\left[SS\right]+\left[SI\right]+\left[ST\right]\right),\\ \left[I\right] & =\frac{1}{k}\left(\left[SI\right]+\left[II\right]+\left[IT\right]\right),\\ \left[T\right] & =\frac{1}{k}\left(\left[ST\right]+\left[IT\right]+\left[TT\right]\right). \end{array}$$

We use this to simplify the model to the six-dimensional pair system (A.1).

### B. Sensitivity Analysis

There is some uncertainty in the numerical values of model parameters; in order to assess the impact of this, we varied *a*, *d*, *r*, and *k* by ± 10%, adjusted *β* to maintain the initial prevalence, and compared model outcomes to our baseline case. Varying the parameters in this way gives sufficiently large coverage of the parameter space, to verify robustness of model outputs. For each parameter varied, we looked separately at increasing screening to 25% per year and also increasing tracing efficiency to 40%.

Figure 5 shows the results for increasing screening coverage to 25% and also for when tracing efficiency is increased to 40%. For each parameter (*a*, *d*, *r*, or *k*), Figure 5 gives the maximum *relative* percentage difference from the baseline values of *I* and *T* after 0, 1, 3, or 5 years. The case at 0 years is included because although the starting prevalence is always 8%, the split of infected persons between the *I* and *T* classes changes with different parameter sets.

Overall, we see that the results are very robust to changes in parameters. The maximum relative difference is around 10%, so if we assume the worst-case scenario, and prevalence is 8%, then this would result in an absolute change in prevalence of 0.8% over 5 years. We see that for *a*, *d*, and *r*, the screening and tracing results are both affected roughly equally, with *r* making the least difference. Changes in the size of *k* have a much larger effect on the results when tracing efficiency is changed than screening coverage (although this is still small). This is as expected because the effectiveness of tracing depends crucially on how many partners each individual has.
